# Views of people with traumatic spinal cord injury about the components of self-management programs and program delivery: a Canadian pilot study

**DOI:** 10.1186/s12883-014-0209-9

**Published:** 2014-10-21

**Authors:** Sarah EP Munce, Michael G Fehlings, Sharon E Straus, Natalia Nugaeva, Eunice Jang, Fiona Webster, Susan B Jaglal

**Affiliations:** Institute of Health, Policy, Management and Evaluation, University of Toronto, 160-500 University Ave, Toronto, Ontario M5G 1 V7 Canada; Department of Surgery, Division of Neurosurgery, University of Toronto, Toronto, Ontario Canada; Li Ka Shing Knowledge Institute of St. Michael’s Hospital, Toronto, Ontario Canada; Department of Applied Psychology & Human Development, Ontario Institute for Studies in Education, Toronto, Ontario Canada; Department of Family and Community Medicine, University of Toronto, Toronto, Canada; Department of Physical Therapy, University of Toronto, Toronto, Canada; Toronto Rehabilitation Institute, Toronto, Canada

**Keywords:** Self-management, Exercise, Nutrition, Depression, Traumatic, Spinal cord injury, Survey

## Abstract

**Background:**

Given the increasing emphasis on the community management of spinal cord injury (SCI), strategies that could be developed and implemented in order to empower and engage individuals with SCI in promoting their health and minimizing the risk of health conditions are required. A self-management program could be one approach to address these complex needs, including secondary complications. Thus, the objective of this study was to determine the importance attributed to the components of a self-management program by individuals with traumatic SCI and explore their views/opinions about the delivery of such a program.

**Methods:**

Individuals with SCI were recruited by email via the Rick Hansen Institute (Vancouver, British Columbia, Canada) as well as an outpatient hospital spinal clinic. Data were collected by self-report using an on-line survey.

**Results:**

The final sample size was 99 individuals with traumatic SCI. The components of a self-management program that were rated as “very important” by the greatest proportion of participants included: exercise (*n=* 53; 53.5%), nutrition (*n=* 51; 51.5%), pain management (*n=* 44; 44.4%), information/education on aging with a SCI (*n=* 42; 42.4%), communicating with health care professionals (*n=* 40; 40.4%), problem solving (*n=* 40; 40.4%), transitioning from rehabilitation to the community (*n=* 40; 40.4%), and confidence (*n=* 40; 40.4%). Overall, 74.7% (*n=* 74) of the sample rated the overall importance of the development of a self-management program for individuals with traumatic SCI as “very important” or “important”. Almost 40% (*n=* 39) of the sample indicated that an internet-based self-management program would be the best delivery format. The highest proportion of participants indicated that the program should have individuals of a similar level of injury (*n=* 74; 74.7%); having individuals of a similar age (*n=* 40; 40.4%) was also noted. Over one-quarter of the sample (*n=* 24) had a depression score consistent with significant symptoms of depression.

**Conclusions:**

Future research is needed to further evaluate how the views of people with traumatic SCI change over time. Our findings could be used to develop and pilot test a self-management program for individuals with traumatic SCI.

**Electronic supplementary material:**

The online version of this article (doi:10.1186/s12883-014-0209-9) contains supplementary material, which is available to authorized users.

## Background

The trend of decreasing length of stay in rehabilitation facilities has led to individuals with spinal cord injury (SCI) entering the community with fewer self-care skills to prevent secondary complications [1,2]. Families and others comprising their informal support network also have less time to adjust. As a result, there is evidence to suggest that these reduced lengths of stay in rehabilitation and associated consequences lead to higher rates of secondary complications and subsequent high rehospitalization rates [3-5]. Furthermore, individuals with a SCI are at particular risk of lifestyle-related diseases including diabetes and heart disease [6]. Given this increasing emphasis on the community management of SCI, strategies that could be developed and implemented in order to empower and engage individuals with SCI in promoting their health and minimizing the risk of health conditions, both those related to their injures and lifestyle-related conditions, are required [7]. A self-management program could be one approach to address these complex needs, including secondary complications.

Hirsche and colleagues [8] conducted a qualitative study on the experiences of individuals with neurological conditions, including stroke, multiple sclerosis, and SCI, who participated in the Stanford Chronic Disease Self-Management Program (CDSMP). The CDSMP is one of the most validated and widely used programs [9,10]. The program is consistent with Bandura’s self-efficacy theory, a social cognitive theory that states that the key predictors of successful behaviour change are confidence (self-efficacy) in the ability to carry out an action and expectation that a particular goal will be achieved (outcome expectancy) [11]. The CDSMP includes the following topics: an overview of self-management principles, exercise, pain and fatigue management, relaxation techniques (e.g., guided imagery and breathing exercises), dealing with depression, nutrition, communicating with family and health professionals, problem solving, and goal setting. The study by Hirsche and colleagues [8] was the first study to examine the experience or the effect of the CDSMP on individuals with SCI. Individuals with SCI as well as the individuals leading the self-management program in this study suggested the need for a SCI-focused group (e.g., individuals with SCI needed information specific to and modules adapted for people dependent on wheelchairs or with limited mobility). In addition, they also found that when attendant care is an important component, as in the case of individuals with SCI, a different approach may be needed to teach self-management skills (i.e., being an effective director of care, instead of a person who manages care independently) [8]. Specific self-management programs have been developed for other chronic diseases including arthritis (e.g., Arthritis Self-Management Program) [12] and stroke (e.g., Moving on After Stroke) [13]. There is a need for both greater understanding of self-management in SCI and detail on the specific components of a self-management program for individuals with SCI. Thus, the objective of this study was to determine the importance attributed to the components of a self-management program by individuals with traumatic SCI and explore their views/opinions about the delivery of such a program. This is the first study of its kind, in Canada or elsewhere, to consider such components, and is the third of a four part study related to factors that should be considered in tailoring self-management programs for individuals with traumatic SCI. These studies are based on the first author’s (SM) doctoral dissertation at the University of Toronto. The first qualitative study on the perceived barriers and facilitators to self-management in individuals with traumatic SCI has been published recently [14].

## Methods

### Study design

A national, cross-sectional study was conducted including individuals with traumatic SCI living in the community in Canada. Research ethics approval was obtained from the University of Toronto (Protocol Reference #26429). All participants provided informed consent prior to completing the survey.

### Participants and recruitment

Eligible participants included individuals who 1) had experienced a traumatic SCI (e.g., a fall, motor vehicle accident, and sporting accidents); 2) were 18 years of age or older; and, 3) were fluent in English. Participants were recruited by email via the Rick Hansen Institute (RHI) and included individuals who had agreed to be contacted for research purposes. RHI is a Canadian-based not-for-profit organization “committed to accelerating the translation of discoveries and best practices into improved treatments for people with SCI” [15]. Additional participants were recruited in person via the outpatient spinal clinic at Toronto Western Hospital. All participants were recruited between January and June 2013.

### Data collection and survey items

Data were collected by self-report surveys using on-line software, FluidSurveys®. The complete survey is included in Additional file 1. The specific content of the survey itself was based on the themes that emerged from the first phase of this study on the facilitators and barriers to self-management in traumatic SCI (i.e., influence of positive/negative mood, self-efficacy) [14] as well as the important psychological elements (i.e., module on depression)/underlying theory of the CDSMP (i.e., social cognitive theory and self-efficacy) [9-11].

Participants were asked to rate the importance of various content modules as well as the overall importance of a self-management program for individuals with traumatic SCI on a Likert-type scale with five response options (1= very unimportant, 5= very important). These modules were based on the themes from our previous, qualitative research on the perceived facilitators and barriers to and meaning of self-management in traumatic SCI (e.g., importance of positive outlook and acceptance/difficulties achieving positive outlook or mood, and maintaining independence/control over care) (e.g., [14]); as well as the existing modules in the CDSMP (e.g., exercise, pain management, fatigue management). In addition, participants were asked to indicate what they thought would be the best modes of delivery (e.g., internet-based, telehealth system, a series of DVDs), format (e.g., one-on-one, in a group setting), construction of program components (e.g., individuals of a similar age, individuals of a similar gender, individuals with a similar level of injury), timing (e.g., during the acute care period, during the rehabilitation period, during the transition from rehabilitation to the community), follow-up periods (e.g., meet again as a group one or two years after the completion of the first program, regular contact with individuals from the group, regular contact with a health care professional), program leader(s) (e.g., health care professional(s) such as a nurse, non-health care professionals, individual(s) who has/have a traumatic SCI), and program organizer(s) (e.g., family physician, physiatrist, case manager).

Standardized questionnaires were also used to capture the qualitative themes generated from the first phase of the study (i.e., [14]) as well as the important elements of the CDSMP. These standardized questionnaires were embedded within the larger survey. These questionnaires included the Hospital Anxiety and Depression Scale (HADS) [16], the short version of the Patient Activation Measure (PAM) [17,18], the Moorong Self-Efficacy Scale (MSES) [19], and the Pearlin-Schooler Mastery Scale (PMS) [20].

### The Hospital Anxiety and Depression Scale (HADS)

The HADS is a brief, self-report measure that was designed to detect the presence and severity of relatively mild degrees of mood disorder in non-psychiatric, hospital outpatients. It provides separate scores for anxiety and depression. The HADS has 14 items (seven for anxiety and seven for depression) and has established validity and reliability (the Cronbach’s alpha for the HADS-Anxiety has a reported mean of 0.83; the HADS-Depression has a reported mean of 0.82) [21]. Scores range from 0 to 21, with higher scores indicating greater symptoms of anxiety and depression [16]. Cut-off scores of ≥8 have been used previously to denote clinically anxious or depressed mood [22].

### The Patient Activation Measure (PAM)

The PAM is designed to assess an individual’s knowledge, skill, and confidence in managing his or her own health care [17,18]. The short version consists of 13 items and uses a Likert-type agreement scale with four response options (1= strongly disagree, 4= strongly agree). The raw score is calculated by adding all of the responses to the 13 questions and range from 13 to 52. These raw scores are converted into activation scores. The converted activation scores range from 0 to 100. Based on these activation scores, individuals are placed into one of four stages of progressive activation: believes active role is important (PAM score of ≤47.0), has the confidence and knowledge to take action (PAM score of 47.1 to 55.1), is taking action (PAM score of 55.2 to 67.0), and is able to stay the course under stress (PAM score of ≥67.1) [17,18,23]. Previous research has demonstrated that higher PAM scores are associated with healthy behaviours, a higher likelihood of performing self-management, and higher medication adherence [24]. Individuals scoring at the lower end of the activation may believe that the physician is the one to “fix” them; mid-range scores may indicate that individuals recognize that they may be involved in their care, but lack the knowledge to do so effectively. Individuals with high PAM scores have gained confidence in their ability to self-manage and make lifestyle changes. The PAM was developed and validated in insured community-based samples in the US [18,23].

### Moorong self-efficacy scale

The MSES was developed to measure an individual’s confidence in performing functional, social, leisure, and vocational activities post-SCI. Participants rate their confidence in their ability to complete the 16 tasks on a seven-point Likert scale (1= very uncertain, 7= very certain). The total scale score is obtained by summing the individual item responses and range from 16 to 112. Higher scores indicate higher perceived self-efficacy. Results have confirmed that the MSES is a valid instrument that is sensitive to real-life changes post-SCI [19].

### Pearlin-schooler mastery scale

The PMS measures global sense of personal control. It consists of seven items and participants respond to a five-point Likert scale about the extent to which they agree (5= strongly agree) or disagree (1= strongly disagree) with the various statements. A PMS score ranges from 7 to 35, with a higher score reflecting greater mastery [20].

In addition, socio-demographic and injury-related variables were documented including age, sex, marital status, level of education, level of injury, and time since injury. Multiple iterations of the survey instrument were produced and reviewed by the research team for flow and content.

### Data analysis

Descriptive statistics on the recruitment of participants, socio-demographic and clinical characteristics (means, medians, and percentages) were calculated. The participants who completed the survey and the individuals who failed to complete the survey were compared according to age, sex, and level of injury using independent *t*-test and Chi-Square test, or Mann–Whitney *U*-test. This was to determine whether the “completion” group was representative of the larger group of eligible individuals identified for the study. The importance of the various content modules of a self-management program, the views related to the delivery, format, construction of program components, follow-up periods, program leader(s), program organizer(s), as well as the overall importance of developing a self-management program have been reported as proportions. To assess how these ratings might relate to the duration of the SCI (i.e., recent - five or less years since the time of injury; longer time since injury - six or more years since the time of injury) Chi-Square tests were conducted. Statistics were calculated using the Statistical Package for the Social Sciences software [25].

## Results

### Response characteristics and sample comparability

The current sample of participants was drawn from the RHI SCI Community database. There were a total of 1417 participants. Of the 1417 participants, 71 did not wish to be contacted for future studies, leaving 1346 participants who did want to be contacted for future studies. A random sample of 300 participants was drawn from this sample of 1346 participants.

Survey invitations were sent to 300 individuals with SCI including individuals with both traumatic and non-traumatic SCI from the RHI; four additional individuals were approached at the Toronto Western Hospital outpatient spinal clinic. The number of individuals with non-traumatic SCI (i.e., and therefore ineligible to participate in the study) was unknown. From this, 145 responded to the survey invitation; with 114 participants completing the entire survey.

Five individuals had injuries of non-traumatic origin and were therefore excluded (109 individuals with traumatic SCI). A further 10 outliers were excluded due to perfect patient activation scores (a measure of self-management behaviour), as per the recommendation of Hibbard and colleagues [17,18] yielding a final sample size of 99 individuals.

There were no significant differences between individuals who completed the survey (*n=* 114) and individuals who did not complete the survey (*n=* 31) in relation to age and gender (*P* >0.05). However, there was a significant difference between group membership (i.e., individuals who completed the survey and individuals who did not complete the survey) and level of injury (Chi Square (2)= 7.915, *P*<0.05).

### Sample characteristics

Selected sociodemographic and clinical characteristics are presented in Table 1. The majority of the sample was male (*n=* 74; 74.7%) and represented a chronic SCI sample with time since injury ranging from<1-54 years (mean= 17.5 years; median= 16 years). Most participants reported having a family physician (*n=* 94; 94.9%). Among those that had a family physician, the most common reason for a family physician visit in the past 12 months was for bladder dysfunction (e.g., urinary tract infection) (*n=* 45; 47.9%), followed by pain (*n=* 35; 37.2%), and bowel issues (*n=* 19; 20.2%). Overall, 88% (*n=* 87) of the sample had visited their family physician for any reason in the previous 12 months. Eleven percent (*n=* 10) of the sample reported that they had visited their family physician in the past 12 months for depression. However, among this group with a family physician, 25.5% of the sample (*n=* 24) had a HAD scale depression score consistent with significant symptoms of depression. A third of the sample (*n=* 33) reported that they had visited the emergency department in the past 12 months; with bladder dysfunction (*n=* 9; 27.3%) cited by the highest proportion of participants, followed by injury (*n=* 6; 18.2%), and pain (*n=* 5; 15.2%). Lastly, in terms of the patient activation levels, 7.1% (*n=* 7) of the sample were in the “starting to take a role”, 9.1% (*n=* 9) were in the “building knowledge and confidence”, 21.2% (*n=* 21) were in the “taking action”, and 62.6% (*n=* 62) were in the “maintaining behaviours” segments. Thirteen percent of participants in the highest activation group had a depression score consistent with significant symptoms of depression . Other psychological characteristics of the sample have been reported in Table 2.

**Table 1:** **Sociodemographic and clinical characteristics of community living individuals with traumatic spinal cord injury**

**Characteristic**	**N= 99**
	**n (%)**
**Age-**Mean, (s.d.), range, median	50.5, (12.1), 55, 52
**Sex**	
Male	74 (74.7)
Female	25 (25.3)
**Relationship status**	
Married	48 (48.5)
Living with a common-law	6 (6.1)
Widowed/Separated/Divorced	20 (20.2)
Single, never married	25 (25.3)
**Education**	
Less than high school	11 (11.1)
High school	25 (25.3)
Trade certificate/College/University certificate or diploma	32 (32.3)
Bachelor’s degree	21 (21.2)
Degree above the Bachelor’s degree	10 (10.1)
Current province*	
Ontario	34 (34.3)
British Columbia	21 (21.2)
Alberta	16 (16.2)
Other	28 (28.3)
**Have children**	
Yes	60 (60.6)
No	39 (39.4)
**Primary caregiver**	
Spouse	26 (26.3)
Attendant	23 (23.2)
No primary caregiver	39 (39.4)
Other	11 (11.1)
**Home setting**	
Home without health services	58 (58.6)
Home with health services	17 (17.2)
Apartment without health services	15 (15.2)
Other	9 (9.1)
**Level of injury**	
Quadriplegia	38 (38.4)
Paraplegia	49 (49.5)
Don’t know	12 (12.1)
Time since injury**-Mean, (s.d.), range, median	17.5, (12.3), 54, 16
**Etiology**	
Sport	19 (19.2)
Fall	15 (15.2)
Transport or motor vehicle collision	50 (50.5)
Other	15 (15.2)
**Brain injury**	
Yes	7 (7.1)
No	88 (88.9)
Don’t know	4 (4.0)

*Representative was achieved from 11 of the 13 provinces and territories in Canada.

**Based on 95 participants.

**Table 2:** **Psychological characteristics of community living individuals with traumatic spinal cord injury**

**Characteristic**	**N= 99**
	**Mean ± SD; n (%)**
**Depression (HADS)**	
Presence of significant symptoms	24 (24.2)
Absence of significant symptoms	75 (75.8)
**Anxiety (HADS)**	
Presence of significant symptoms	32 (32.3)
Absence of significant symptoms	67 (67.7)
**Activitation Level (PAM)**	
Starting to take a role	7 (7.1)
Buidling knowledge and confidence	9 (9.1)
Taking action	21 (21.2)
Maintaining behaviours	62 (62.6)
**Self-efficacy (Total MSES)**	87.8 ± 18.6
**Mastery (Total PMS)**	21.9 ± 3.9

### Components of a self-management program

The ratings for all of the content modules and overall importance of a self-management program are reported in Table 3. The other components including modes of delivery, format, construction of program components, timing, follow-up periods, program leader(s), and program organizer(s) have been reported in Table 4. Based on all of these findings, a summary of the proposed components of a self-management program for individuals with traumatic SCI is presented in Table 5. Some of the components of a self-management program that were rated as “very important” by the greatest proportion of participants included: exercise (*n=* 53; 53.5%), nutrition (*n=* 51; 51.5%), pain management (*n=* 44; 44.4%), and confidence (in reducing secondary complications/promoting wellness) (*n=* 40; 40.4%). Overall, 74.7% (*n=* 74) of the sample rated the overall importance of the development of a self-management program for individuals with traumatic SCI as “very important” or “important”.

**Table 3:** **Importance attributed to the components of a self-management program by community living individuals with traumatic spinal cord injury**

**Component**	**Very unimportant**	**Unimportant**	**Neither important or unimportant**	**Important**	**Very important**
	**n (%)**	**n (%)**	**n (%)**	**n (%)**	**n (%)**
Exercise	1 (1.0)	2 (2.0)	2 (2.0)	41 (41.4)	53 (53.5)
Pain management	1 (1.0)	5 (5.1)	8 (8.1)	41 (41.4)	44 (44.4)
Fatigue management	0 (0.0)	5 (5.1)	16 (16.2)	50 (50.5)	28 (28.3)
Relaxation techniques	5 (5.1)	9 (9.1)	44 (44.4)	32 (32.3)	9 (9.1)
Dealing with depression	5 (5.1)	6 (6.1)	12 (12.1)	42 (42.4)	34 (34.3)
Nutrition	2 (2.0)	3 (3.0)	0 (0.0)	43 (43.4)	51 (51.5)
Communicating with family	2 (2.0)	1 (1.0)	17 (17.2)	43 (43.4)	36 (36.4)
Communicating with HCPs	2 (2.0)	2 (2.0)	8 (8.1)	47 (47.5)	40 (40.4)
Problem solving	3 (3.0)	1 (1.0)	14 (14.1)	41 (41.4)	40 (40.4)
Goal setting/action planning	1 (1.0)	4 (4.0)	23 (23.2)	47 (47.5)	24 (24.2)
Information/education on aging with a SCI	1 (1.0)	1 (1.0)	10 (10.1)	45 (45.5)	42 (42.4)
Information/education on sexuality and SCI	2 (2.0)	5 (5.1)	24 (24.2)	50 (50.5)	18 (18.2)
Relationship issues (e.g., with your spouse)	2 (2.0)	4 (4.0)	20 (20.2)	41 (41.4)	32 (32.3)
Confidence	2 (2.0)	1 (1.0)	13 (13.1)	43 (43.4)	40 (40.4)
Decision making abilities	2 (2.0)	3 (3.0)	13 (13.1)	47 (47.5)	34 (34.3)
Can provide mentorship opportunities	2 (2.0)	3 (3.0)	35 (35.4)	43 (43.4)	16 (16.2)
Can receive mentorship opportunities	3 (3.0)	3 (3.0)	35 (35.4)	40 (40.4)	18 (18.2)
Learning about volunteer opportunities	2 (2.0)	9 (9.1)	48 (48.5)	29 (29.3)	11 (11.1)
Skills to enter/re-enter to job market	4 (4.0)	6 (6.1)	20 (20.2)	36 (36.4)	33 (33.3)
Issues of transitioning from rehabilitation to the community	4 (4.0)	3 (3.0)	15 (15.2)	37 (37.4)	40 (40.4)
Overall importance	25 (25.3)	0 (0.0)	0 (0.0)	34 (34.3)	40 (40.4)

*Abbreviations:* *SCI* Spinal Cord Injury, *HCP* Health Care Professional.

**Table 4:** **Views of community living individuals with traumatic spinal cord injury about self-management program delivery**

**Component**	**N= 99**
	**n (%)**
**Mode of delivery**	
Internet-based	39 (39.4)
A series of DVDs	7 (7.1)
In person, in the community	30 (30.3)
Other (telehealth system, brochure, by telephone, etc.)	23 (23.2)
**Format**	
One-on-one (i.e., one facilitator to one client)	29 (29.3)
Individually (e.g., the client views a webinar individually)	22 (22.2)
In a group setting with other individuals with traumatic spinal cord injury	10 (10.1)
In a group setting with other individuals with traumatic spinal cord injury together with their caregivers	9 (9.1)
In a group setting with other individuals with traumatic spinal cord injury together with their caregivers, but with opportunities for separate discussions	21 (21.2)
Other	8 (8.1)
**Construction of program components**	
Similar age (=yes)	40 (40.4)
Same gender (=yes)	29 (29.3)
Similar level of injury (=yes)	74 (74.7)
Individuals with non-traumatic SCI (=yes)	26 (26.3)
Individuals in a wheelchair (=yes)	28 (28.3)
Other (=yes)	16 (16.2)
**Timing**	
During the acute care period	7 (7.1)
During the rehabilitation period	42 (42.4)
During the transition from rehabilitation to the community	29 (29.3)
Once accustomed to living in the community	11 (11.1)
Other	10 (10.1)
**Need for follow-up sessions or program**	
Yes	88 (88.9)
No/Don’t know	11 (11.1)
Follow-up periods***	
Meet again as a group 1 or 2 years after	18 (18.2)
Regular contact with an individual(s) from the group	25 (25.3)
Regular contact with a health care professional	31 (31.3)
Other	16 (16.2)
**Program leaders**	
Health care professional (e.g., nurse, rehabilitation specialist)	38 (38.4)
Individual(s) who has/have a traumatic spinal cord injury	19 (19.2)
Combination of any of the choices above	38 (38.4)
Other (e.g., non-health care professionals, individual(s) who has/have a neurological conditions)	4 (4.0)
**Program organizers**	
Family physician	3 (3.0)
Physiatrist	3 (3.0)
Case manager	4 (4.0)
Staff in the acute care team	1 (1.0)
Staff in the rehabilitation team	37 (37.4)
An organization such as the Canadian Paraplegic Association	41 (41.4)
Staff in the home care team	2 (2.0)
Other	8 (8.1)

***Based on 90 participants.

**Table 5:** **Summary of the views of community living individuals with traumatic spinal cord injury about the components of a self-management program and program delivery**

**Component**	**Description**
Content modules	-Exercise
	-Nutrition
	-Pain management
	-Communicating with health care professionals (e.g., mood) and/or directing care
	-Dealing with depression
	-Problem solving
	-Confidence (i.e., self-efficacy)
	-(Preventing) bowel/bladder dysfunction
	-(Preventing) injury
Mode of delivery	Internet-based
Format	One-on-one (i.e., one facilitator to one client)
Construction of program components	Individuals with a similar level of injury, age
Timing	Rehabilitation or transition from rehabilitation to the community periods
Follow-up periods	Occurring as regular contact with a health care professional and/or regular contact with an individual(s) from the group
Program leaders	Health care professional and an individual with a traumatic SCI
Program organizers	Organization such as Spinal Cord Injury Canada or rehabilitation team

In terms of modes of delivery, 39.4% (*n=* 39) of the sample thought that an internet-based self-management program would be best, and within this mode of delivery, 29.3% (*n=* 29) selected a one-on-one format (i.e., one facilitator to one client). In terms of construction of program components, the highest proportion of participants thought that having individuals with a similar level of injury (*n=* 74; 74.7%) would be best; having individuals of a similar age (*n=* 40; 40.4%) was also noted. The rehabilitation period (*n=* 42; 42.4%) and the transition from rehabilitation to the community (*n=* 29; 29.3%) were viewed as the best times to introduce a self-management program. The majority of the sample (*n=* 88; 88.9%) responded positively about the need for a follow-up session or a follow-up program; 31.3% (*n=* 31) thought that the follow-up periods occurring as regular contact with a health care professional would be helpful. One quarter (*n=* 25; 25.3%) of the sample thought that having the follow-up periods occurring as regular contact with a(n) individual(s) from the group would be helpful. In terms of program leaders, 38.4% (*n=* 38) thought that a health care professional and an individual with a traumatic SCI should deliver the self-management program. Forty-one percent (*n=* 41) indicated that an organization such as SCI Canada (a community-based service provider to individuals living with SCI) should organize the self-management program; 37.4% indicated that the rehabilitation team should organize the program. Lastly, in terms of variation by time since injury status and the components of the self-management program, individuals with a longer time since injury were more likely to rate the information/education on aging with a SCI and the relationship issues modules as important/very important compared to their counterparts with a more recent time since injury (Chi Square (2)= 7.521, *P*<0.05; Chi Square (2)= 6.305, *P*<0.05, respectively). No other differences emerged in terms of time since injury status and the components of the self-management program.

## Discussion

The objective of the current study was to determine the importance attributed to the components of a self-management program by individuals with traumatic SCI including the importance attributed to various content modules and other characteristics including delivery, format, and construction of program components. To the best of our knowledge this is the first study, in Canada or elsewhere, to examine the views of individuals with traumatic SCI about the components of a self-management program and is one of only a few studies to consider the consumer perspective in the development of a self-management program.

Almost one quarter of the sample had a HAD scale depression score consistent with significant symptoms of depression. Previous research has indicated that major depression is the most common psychological condition associated with SCI, affecting approximately 25% to 30% of individuals with SCI living in the community [26]. In a review of psychosocial issues in SCI, Post and van Leeuwen [27] indicated that six studies had examined anxiety in SCI [28-33]. Clinically significant symptoms of anxiety in SCI have been previously reported as ranging from 13.2% to 40%. To the best of our knowledge, this is the first study to use the PAM in a sample of individuals with SCI. Almost two thirds of the current sample were in the “maintaining behaviours” segment of patient activation/self-management. This proportion was inconsistent with previous findings from a national (US) sample on individuals 45 years and older, demonstrating 22% were in the “maintaining behaviours” segment of patient activation/self-management [18,23]. Rask and colleagues [34] similarly determined that 62% of their participants were in the highest stage of activation in their study of underprivileged individuals with diabetes. Consistent with the conclusions of Rask and colleagues [34], future research is needed to confirm the findings related to patient activation in the present study, and explore the use of adapted activation measures that might better differentiate levels of readiness for self-management among individuals with (traumatic) SCI, specifically. Lastly, there has been a lack of studies on mastery in SCI. Lannem and colleagues [35] demonstrated that perception of exercise mastery was negatively related to exercise in persons with incomplete SCI, in contrast to those with complete lesions. Kinder [36] previously outlined a model of ‘hardiness’ in SCI. This model included taking responsibility for decisions (control), making a commitment to choices (commitment), and creating alternatives to problems (challenge). It was determined that in those demonstrating (high) ‘hardiness’, control was associated with making decisions based on choices of care, commitment was associated with achieving goals and maintaining independence, and challenge was associated with solving problems and attaining mastery [36]. Mastery experience (i.e., actual performance of a behavior or task) is believed to be the most powerful source of information influencing self-efficacy [11]. Thus, future research should continue to explore the role of mastery in SCI and its impact on other mental health outcomes.

Many of the content modules that were rated as important for a self-management program were existing modules of the CDSMP, as outlined above, including exercise, nutrition, pain management, communicating with health care professionals, problem solving, and confidence. At the same time, the importance assigned to the modules on exercise and nutrition in the current study is in line with our previous, qualitative research on the meaning of self-management in traumatic SCI. Our research determined that part of the meaning of self-management according to individuals with traumatic SCI and their (mainly family) caregivers included the notion of “wellness awareness” which included lifestyle practices/changes including good nutrition, vitamin supplementation, exercise, and relaxation that these participants associated with living well and maintaining/optimizing health [unpublished observations; Munce, Webster, Fehlings, Straus, Jang, & Jaglal]. Wellness/health promotion interventions (i.e., focusing on exercise and nutrition) are resources that allow the individual to choose behaviours to enhance and sustain quality of life within the context of living with a chronic disabling condition [37], and thus could increase the individual’s sense of control over his or her care (i.e., self-efficacy). Healthier lifestyles are unequivocally linked to better health care outcomes as well as better health and reduced health risks and potentially life-saving for these individuals.

Approximately 40% of the current sample indicated that a module on increasing confidence was “extremely important”, a finding which is consistent with the importance/centrality of self-efficacy in the CDSMP [9,10]. We also previously determined the importance of the individual with SCI maintaining control over care as an important facilitator to self-management in SCI [14]. Furthermore, more than three-quarters of the sample rated the inclusion of a module on “dealing with depression” as “very important” or “important”. This finding is consistent with our previous findings on the importance of positive and negative mood as either a facilitator or barrier to self-management [14]. The fact that one quarter of the current sample had symptoms of depression and yet only ten percent of the sample had visited their family physician for their depression/symptoms of depression may be related to the stigma of mental illness/seeking help for mental illness [38]. Furthermore, given the fact that the “communicating with health care professionals” module was rated as important, this finding may relate to a difficulty introducing the topic of mood/depression, specifically, with a health care professional and a desire for some skills/confidence to introduce the topic. Moreover, the low rate of family physicians visits related to depression found in the current study has been previously identified and may be because the ongoing physical/medical concerns of individuals with SCI are given priority over the emotional concerns of individuals with SCI [38]. Overall, more research is needed to examine the barriers to care that may be contributing to the low rates of depression treatment in SCI as well as to systematically study treatments for depression (e.g., coping effectiveness training) after SCI [39].

A module on “information/education on aging with a SCI” was also rated as an important module. As progress in health care and rehabilitation treatment has improved, individuals with more complex needs, including chronic conditions such as diabetes and heart disease, are surviving [6]. Indeed, individuals with a longer time since their injury were more likely to rate this module as important/very important as compared to their counterparts with a more recent time since injury. This finding likely reflects an emerging relevance of the module. Aging with a SCI was found to be a cross-cutting theme in our previous, qualitative research: aging/chronic conditions were found to be a complicating factor in maintaining positive physical (i.e., secondary complications) and mental health (i.e., mood) [14]. Based on the most commonly cited reasons for a family physician/emergency department visit, modules on (preventing) bowel/bladder dysfunction and injury are recommended, although these were not included as module/content response options on the survey. Individuals with a longer time since injury were also more likely to rate the relationship issues module as important/very important compared to their counterparts with a more recent time since injury. It is likely that once individuals with traumatic SCI become experienced/expert in managing their health (i.e., monitoring for secondary complications), they can then turn their attention to/are motivated to work on their relationship issues. Lastly, while the majority of the sample rated the overall importance of the development of a self-management program for individuals with traumatic SCI as “very important” or “important”, one-quarter of the sample indicated that the development of a self-management program was “very unimportant”. This finding may reflect health-system level barriers including, access and availability of services and models of care, which limit the ability to create the optimal conditions for self-management among individuals with traumatic SCI (i.e., a self-management program is not viewed as important if the health system does not have the characteristics needed to support the individual’s self-management) [14]. Future studies should aim to understand the reasons why self-management may not be considered as important among particular sub-groups of individuals with traumatic SCI.

The greatest proportion of participants thought that an internet-based self-management program would be best. Internet-based applications/tools are recognized as having the potential to overcome barriers to self-management skills including cost and access (e.g., delivery independent of time and location) [40]. We previously highlighted lack of accessibility as one of the barriers to self-management (e.g., accessing buildings, difficulties with accessing physician offices and/or exam tables, distance/transportation) [14], and thus the view that an internet-based program would be the best delivery format for a self-management program is consistent with the need to address barriers with respect to accessibility. On-line self-management programs have previously demonstrated improvements in self-efficacy and patient activation when compared to usual care e.g., [41].

Almost one third of participants thought that a one-on-one format (i.e., one facilitator to one client, in real time) would be best, which may reflect a need/desire for more intensive/individualized support given the potential physical (e.g., secondary complications) and emotional complexities of living with a SCI. At the same time, group programs include the benefit of peer support and opportunities for interaction [42]. Almost 40% of participants thought that both a health care professional and an individual with a SCI should deliver the program. This is not unlike other self-management programs which can be led by both a health care professional and a trained peer leader who has the same condition as the participants. Again, this view may reflect a desire to address both the physical (i.e., via the health care professional) and emotional (i.e., via the peer leader) complexities of a SCI. In terms of construction of program components, almost 75% of participants indicated that having individuals with a similar level of injury would best suit their needs. A program composed of individuals of a similar age was also noted. The importance of this matching has been recognized in previous studies whereby demographic/clinical information such as age, race, and etiology of injury was considered when assigning a potential mentee to a mentor [43]. While some of these findings may seem incongruous, this combination of format and construction of program components is consistent with the internet-based CDSMP that Lorig and colleagues [41] have implemented in diabetes. For example, participants are able to log on individually to sessions and can use a “Post Office” where they are able to write individual messages to a facilitator. At the same time, participants are asked to respond to weekly questions (e.g., “What problems do you have because of your diabetes?) and make a specific action plans. The questions and action plans are posted on the bulletin boards in the Discussion Centre, where they can be seen by all participants. Thus, based on the present findings, the format and construction of program components, as described by the internet-based CDSMP in diabetes, may be relevant to individuals with traumatic SCI - they could use the “Post-Office” for a one-on-one interaction with the facilitator, but at the same time, think that having individuals of a similar level of injury would be best for interactions in the Discussion Centre. The rehabilitation or the transition from rehabilitation to the community period were viewed as the best times to introduce a self-management program. Given the importance of motivation/readiness to change in self-management [42,44,45], this represents a key implementation consideration. Lastly, the majority of participants thought that an organization such as SCI Canada should be responsible for organizing the program. Given that SCI Canada already organizes a peer support counseling program, some existing infrastructure (staff, peer mentors) could likely be used for the organization of a self-management program.

The current study acknowledges some limitations, especially with respect to the generalizability of our study findings. Although the sampling frame was national, the resulting sample was small and may not be representative of the population of people with traumatic SCI in Canada. Thus, it is likely that it was not representative of the broader group of individuals with traumatic SCI. Instead the current sample likely represented a more engaged and healthier group of individuals with traumatic SCI (e.g., 95% of the sample reported having a family physician; 62.6% were in the “maintaining behaviours” segment of self-management/activation; all participants wished to be contacted for research purposes). The generalizability of the study findings to individuals with non-traumatic SCI is unknown, although the study findings may be limited to individuals with traumatic SCI as individuals with non-traumatic SCI tend to be older and include more females [46]. Thus, this may mean, for example, that fewer individuals with non-traumatic SCI would assign importance to a module on exercise (i.e., older individuals would likely have more comorbidities and could be more interested in the module on aging with a SCI; females with SCI may already be more comfortable than their male counterparts in terms of speaking with their family members and/or a health care professional and therefore assign less importance to these modules). Furthermore, the management of SCI varies globally [47], and thus, the current results may be specific to the Canadian context. The design of the current study is cross-sectional and therefore it is unknown how these perspectives on the components of a self-management program change over time. Future research should explore this in greater detail.

## Conclusions

The current study is, to the best of our knowledge, the first of its kind, in Canada or elsewhere, to determine the importance attributed to components of a self-management program by individuals with traumatic SCI. Many of the modules of a self-management program that were rated as important were existing modules of the CDSMP. At the same time, the importance assigned to the modules on exercise and nutrition is in line with our previous, qualitative research and a wellness/health promotion approach for self-management in this population e.g., [14]. Various implementation considerations were determined including an internet-based delivery format, likely to address issues of accessibility. Future research is needed to further evaluate how the views of people with traumatic SCI change over time. Our findings could be used to develop and pilot test a self-management program for individuals with traumatic SCI.

## Additional file

Additional file 1:
**Survey on Considerations for Self-Management Support for Individuals with Traumatic Spinal Cord Injury.**

## Abbreviations

CDSMP: Chronic Disease Self-Management Program
HADS: Hospital Anxiety and Depression Scale
PAM: Patient Activation Measure
RHI: Rick Hansen Institute
SCI: Spinal cord injury
