# Supplementary material for: Views of people with traumatic spinal cord injury about the components of self-management programs and program delivery: a Canadian pilot study
Source: BMC Neurol. 2014 Oct 21;14:209. doi: 10.1186/s12883-014-0209-9 (PMC4210500; doi:10.1186/s12883-014-0209-9)
Supplement: Additional file 1: — Survey on Considerations for Self-Management Support for Individuals with Traumatic Spinal Cord Injury. [file 12883_2014_209_MOESM1_ESM.docx]

**Additional file 1**

Survey on Considerations for Self-Management Support for Individuals with Traumatic Spinal Cord Injury

This study is being carried out as part of the doctoral (PhD) work of Sarah Munce. We will be speaking with approximately 100 individuals with traumatic spinal cord injury. We will be speaking with individuals across Canada. Self-management support may be defined as the systematic provision of education and supportive interventions to increase patients’ skills and confidence in managing their health problems, including regular assessment of progress and problems, goal setting, and problem-solving support. Therefore, we are interested in the specific components of a self-management support program for individuals with a traumatic spinal cord injury – What should the content of this program be? Who should deliver the program (or intervention)? Who should you take the program with? Information collected from this study will lead to a better understanding of the self-care behaviours among individuals with traumatic spinal cord injury, such as yourself. It is hoped that this information will lead to the development of a self-management support program specifically targeted to individuals with traumatic spinal cord injury.You are eligible to complete this survey if you haveall three of the following characteristics:□You sustained your injury because of a traumatic cause (i.e., a fall, motor vehicle collision, sporting accident, etc);□You are 18 years of age or older; and,□You can speak English.If you have any questions or concerns regarding the survey, you can contact Sarah Munce at sarah.munce@utoronto.ca or toll-free at 1-855-946-7902.This survey will take about 30 minutes to complete. Upon completion of the survey, you will be sent a $10.00 gift certificate.

**Consent**

|  | I AGREE to participate in this research study |
| --- | --- |
|  | I REFUSE to participate in this research study |

**PART I – Background and Descriptive Information**

**1. What is your age?**

**2. What is your sex?**

|  | Male |
| --- | --- |
|  | Female |
|  | Other, please specify... ______________________ |

**3. What is your marital status?**

|  | Married |
| --- | --- |
|  | Living common-law |
|  | Widowed |
|  | Separated |
|  | Divorced |
|  | Single, never married |

**4. What racial or cultural group do you belong to?**

|  | White |
| --- | --- |
|  | South Asian (e.g., East Indian, Pakistani, Sri Lankan, etc.) |
|  | Chinese |
|  | Black |
|  | Filipino |
|  | Latin American |
|  | Arab |
|  | Southeast Asian (e.g., Vietnamese, Cambodian, Malaysian, Laotian, etc.) |
|  | West Asian (e.g., Iranian, Afghan, etc.) |
|  | Korean |
|  | Japanese |
|  | Other, please specify... ______________________ |
|  | Unknown |

**5. What is the highest degree, certificate or diploma you have completed?**

|  | Less than high school diploma or its equivalent |
| --- | --- |
|  | High school diploma or a high school equivalency certificate |
|  | Trade certificate or diploma |
|  | College, CEGEP or other non-university certificate or diploma (other than trades certificates or diplomas) |
|  | University certificate or diploma below the bachelor’s level |
|  | Bachelor’s degree (e.g., B.A., B.Sc., LL.B.) |
|  | University certificate, diploma or degree above the bachelor’s level |

**6. What province do you currently live in?**

|  | Newfoundland and Labrador |
| --- | --- |
|  | Prince Edward Island |
|  | New Brunswick |
|  | Quebec |
|  | Ontario |
|  | Manitoba |
|  | Saskatchewan |
|  | Alberta |
|  | British Columbia |
|  | Yukon |
|  | The Northwest Territories |
|  | Nunavut |
|  | Nova Scotia |

**7. What is the size of your community?**

|  | One million or more people |
| --- | --- |
|  | 250,000 to 999,999 people |
|  | 50,000 to 249,999 people |
|  | 20,000 to 49,999 people |
|  | 2,500 to 19,999 people |
|  | 2,499 people or less |
|  | Don’t know |

**8a. Do you have children?**

|  | Yes |
| --- | --- |
|  | No |

**8b. If YES: How many children do you have?**

**9. Who are you currently living with? (check all that apply)**

|  | Partner/spouse |
| --- | --- |
|  | Family member(s) |
|  | Paid attendant |
|  | Alone |
|  | Other, please specify... ______________________ |

**10. Who would you identify as your primary caregiver? (check one response only)**

|  | Spouse |
| --- | --- |
|  | Sibling |
|  | Parent(s) |
|  | Son or Daughter |
|  | Nurse |
|  | Attendant |
|  | I don’t have a primary caregiver |
|  | Other, please specify... ______________________ |

**11. What type of setting do you currently live in? (check one response only)**

|  | Home without health services |
| --- | --- |
|  | Home with health services |
|  | Apartment without health services |
|  | Apartment with health services |
|  | Assisted living facility |
|  | Other, please specify... ______________________ |

**12. What is the level of your injury?**

|  | Cervical |
| --- | --- |
|  | Thoracic |
|  | Lumbar |
|  | Don’t know |

**13. Is your injury complete or incomplete?**

|  | Complete |
| --- | --- |
|  | Incomplete |
|  | Don’t know |

**14. Are you a person with**

|  | Quadriplegia |
| --- | --- |
|  | Paraplegia |
|  | Don’t know |

**15. When did your injury occur (i.e., month, year)?**

**16. How did your injury occur?**

|  | Sport |
| --- | --- |
|  | Assault (blunt) |
|  | Assault (penetrating) |
|  | Fall |
|  | Transport or Motor Vehicle Collision |
|  | Other, please specify... ______________________ |
|  | Unspecified or unknown |

**17. Did you have a traumatic brain injury as a result of your injury in addition to your spinal cord injury?**

|  | Yes |
| --- | --- |
|  | No |
|  | Don’t know |

**Contact with Health Professionals/Health Care Utilization**

Now I’d like to ask you about your contacts with health professionals during the past 12 months, that is, from [date one year ago] to yesterday.

**18a. Do you have a family physician?**

|  | Yes |
| --- | --- |
|  | No |
|  | Don’t know |

**18b. If NO: Why do you not have a family physician? (check all that apply and then SKIP TO 18d)**

|  | No family physicians available in the area |
| --- | --- |
|  | Family physicians in the area are not taking new patients |
|  | Have not tried to contact one |
|  | Had a family physicians who left or retired |
|  | Other, please specify... ______________________ |

**18c. In the past 12 months, have you seen your family physician due to any of the following health problems? (check all that apply)**

|  | Problems with sudden bouts of high blood pressure, intense sweating (i.e., autonomic dysreflexia) |
| --- | --- |
|  | Pressure ulcers (i.e., decubitus ulcers) |
|  | Bladder dysfunction (e.g., urinary tract infection(s)) |
|  | Pneumonia |
|  | Low blood pressure |
|  | Bowel issues |
|  | Blood clots in the leg (i.e., deep venous thrombosis (DVT)) |
|  | Blood clots in the lung (i.e., deep venous thrombosis (DVT)) |
|  | Depression |
|  | Pain |
|  | Injury (e.g., fracture) |
|  | Other, please specify... ______________________ |
|  | Don't know |

**18d. In the past 12 months, what specialist physicians have you seen? (check all that apply)**

|  | Physiatrist |
| --- | --- |
|  | Neurologist |
|  | Urologist |
|  | Neurosurgeon |
|  | Other, please specify... ______________________ |
|  | None; If NO: SKIP TO 18f |
|  | Don’t know |

**18e. In the past 12 months, have you seen your specialist physician(s) due to any of the following health problems (check all that apply)?**

|  | Problems with sudden bouts of high blood pressure, intense sweating (i.e., autonomic dysreflexia) |
| --- | --- |
|  | Pressure ulcers (i.e., decubitus ulcers) |
|  | Bladder dysfunction (e.g., urinary tract infection(s)) |
|  | Pneumonia |
|  | Low blood pressure |
|  | Bowel issues |
|  | Blood clots in the leg (i.e., deep venous thrombosis (DVT)) |
|  | Blood clots in the lung (i.e., deep venous thrombosis (DVT)) |
|  | Depression |
|  | Pain |
|  | Injury (e.g., fracture) |
|  | Other, please specify... ______________________ |
|  | Don't know |

**18f. In the past 12 months, have you visited the emergency department?**

|  | Yes |
| --- | --- |
|  | No; If NO: SKIP TO 18h |
|  | Don’t know |

**18g. In the past 12 months, have you visited the emergency department due to any of the following health problems? (check all that apply)**

|  | Problems with sudden bouts of high blood pressure, intense sweating (i.e., autonomic dysreflexia) |
| --- | --- |
|  | Pressure ulcers (i.e., decubitus ulcers) |
|  | Bladder dysfunction (e.g., urinary tract infection(s)) |
|  | Pneumonia |
|  | Low blood pressure |
|  | Bowel issues |
|  | Blood clots in the leg (i.e., deep venous thrombosis (DVT)) |
|  | Blood clots in the lung (i.e., deep venous thrombosis (DVT)) |
|  | Depression |
|  | Pain |
|  | Injury (e.g., fracture) |
|  | Other, please specify... ______________________ |
|  | Don't know |

**18h. In the past 12 months, have you been admitted to hospital (i.e., been a patient overnight in a hospital)?**

|  | Yes |
| --- | --- |
|  | No If NO: SKIP TO 19a |
|  | Don’t know |

**18i. In the past 12 months, have you been admitted to hospital due to any of the following health problems? (check all that apply)**

|  | Problems with sudden bouts of high blood pressure, intense sweating (i.e., autonomic dysreflexia) |
| --- | --- |
|  | Pressure ulcers (i.e., decubitus ulcers) |
|  | Bladder dysfunction (e.g., urinary tract infection(s)) |
|  | Pneumonia |
|  | Low blood pressure |
|  | Bowel issues |
|  | Blood clots in the leg (i.e., deep venous thrombosis (DVT)) |
|  | Blood clots in the lung (i.e., deep venous thrombosis (DVT)) |
|  | Depression |
|  | Pain |
|  | Injury (e.g., fracture) |
|  | Other, please specify... ______________________ |
|  | Don't know |

**Health Care System Satisfaction**

Now, a few questions about health care services you have received in your province.

**19a. Overall, how would you rate the availability of health care services in your province? Would you say it is:**

|  | Excellent |
| --- | --- |
|  | Good |
|  | Fair |
|  | Poor |
|  | Don’t know |

**19b. Overall, how would you rate the availability of health care services in your community?**

|  | Excellent |
| --- | --- |
|  | Good |
|  | Fair |
|  | Poor |
|  | Don’t know |

**19c. Overall, how would you rate the quality of the health care services that are available in your community?**

|  | Excellent |
| --- | --- |
|  | Good |
|  | Fair |
|  | Poor |
|  | Don’t know |

**PART II – Components of a Targeted Self-Management Support Program**

**1. How would you rate the following items in terms of their importance in a self-management support program to reduce health problems associated with spinal cord injury and promote wellness?**

|  | Very Unimportant | Unimportant | Neither Important or Unimportant | Important | Very Important |
| --- | --- | --- | --- | --- | --- |
| Exercise |  |  |  |  |  |
| Pain management |  |  |  |  |  |
| Fatigue management |  |  |  |  |  |
| Relaxation techniques (e.g., guided imagery and breathing exercises) |  |  |  |  |  |
| Dealing with depression |  |  |  |  |  |
| Nutrition |  |  |  |  |  |
| Communicating with family |  |  |  |  |  |
| Communicating with health care professionals |  |  |  |  |  |

**2. How would you rate the following other items in terms of their importance in a self-management support program to reduce health problems associated with spinal cord injury and promote wellness?**

|  | Very Unimportant | Unimportant | Neither Important or Unimportant | Important | Very Important |
| --- | --- | --- | --- | --- | --- |
| Information/education on aging with a spinal cord injury |  |  |  |  |  |
| Information/education on sexuality and spinal cord injury |  |  |  |  |  |
| Relationship issues (e.g., with your spouse) |  |  |  |  |  |
| Confidence |  |  |  |  |  |
| Decision-making abilities |  |  |  |  |  |
| Can provide mentorship opportunities |  |  |  |  |  |
| Can receive mentorship opportunities |  |  |  |  |  |
| Learning about volunteer opportunities |  |  |  |  |  |
| Skills to enter/re-enter to job market |  |  |  |  |  |
| Issues of transitioning from rehabilitation to the community |  |  |  |  |  |

**3. Are there other components that could be added to a self-management support program to reduce health problems associated with spinal cord injury and promote wellness?**

**4. How would you rate the following items in terms of their importance in a self-management support program for your family member/caregiver?**

|  | Very Unimportant | Unimportant | Neither Important or Unimportant | Important | Very Important |
| --- | --- | --- | --- | --- | --- |
| Information/education on aging with a spinal cord injury |  |  |  |  |  |
| Information/education on sexuality and spinal cord injury |  |  |  |  |  |
| Relationship issues |  |  |  |  |  |
| Specific emotional support for caregivers/family members themselves |  |  |  |  |  |
| Practical support for caregivers/family members themselves (e.g., learning skills to assist you with bladder/bowel management) |  |  |  |  |  |

**5. Are there other components that could be added to a self-management support program to reduce health problems associated with spinal cord injury and promote wellness for your family member/caregiver?**

**6. What do you think would be the best delivery format for a self-management support program? (check one response only)**

|  | Internet-based (e.g., webinar, blog entries) |
| --- | --- |
|  | Telehealth system |
|  | (A series of) DVDs |
|  | Brochure |
|  | In person, in the community (e.g., such as a church hall) |
|  | By telephone |
|  | Other, please specify... ______________________ |

**7. Thinking about the best delivery format for a self-management support program that you selected above, with whom do you think the program would be best delivered? (check one response only)**

|  | One-on-one (i.e., one facilitator to one client) |
| --- | --- |
|  | Individually (e.g., the client views a webinar individually) |
|  | In a group setting with other individuals with traumatic SCI |
|  | In a group setting with other individuals with traumatic SCI together with their caregivers/family members |
|  | In a group setting with other individuals with traumatic SCI together with their caregivers/family members, but with opportunities for individuals with SCI and their caregivers/family members to have separate discussions |
|  | Other, please specify... ______________________ |

**8. In order to best suit your needs, what do you think are some other considerations for the make-up of the self-management support program? (check all that apply)**

|  | The program should have individuals of a similar age |
| --- | --- |
|  | The program should have individuals of the same gender |
|  | The program should have individuals with a similar level of injury |
|  | The program could have individuals with non-traumatic spinal cord |
|  | The program could have individuals in a wheelchair (i.e., they may have another neurological condition, but they don’t necessarily have a spinal cord injury) |
|  | Other, please specify... ______________________ |

**9. In your opinion, what do you think would be the optimal number of sessions for a self-management support program?**

**10. In your opinion, how long do you think each session should last (in hours)?**

**11. In your opinion, when would be the best time to introduce or start a self-management support program for individuals with a traumatic spinal cord injury? Thinking about this another way, and based on your own experience, when would you have been ready to have first received a self-management support program? (check one response only)**

|  | During the acute care period (i.e., shortly after the injury, in hospital) |
| --- | --- |
|  | During the rehabilitation period |
|  | During the transition from rehabilitation to the community |
|  | Once accustomed to living in the community (e.g., ≥ 18 months after the injury) |
|  | Other, please specify... ______________________ |

**12. Again, thinking about your own experiences and your recovery trajectory, at what point(s) would a self-management support program have been useful/helpful (check all that apply)?**

|  | During the acute care period (i.e., shortly after the injury, in hospital) |
| --- | --- |
|  | During the rehabilitation period |
|  | During the transition from rehabilitation to the community |
|  | Once accustomed to living in the community (e.g., ≥ 18 months after the injury) |
|  | Other, please specify... ______________________ |

**13a. At the end of a self-management support program, do you think it would be helpful to have a follow-up self-management session(s) or program?**

|  | Yes |
| --- | --- |
|  | No |
|  | Don’t know |

**13 b. If YES: What form would this follow up take?**

|  | Meet again as a group 1 or 2 years after the completion of the first program |
| --- | --- |
|  | Regular contact with an individual(s) from the group (e.g., another individual with a spinal cord injury) |
|  | Regular contact with a health care professional, such as a nurse |
|  | Other, please specify... ______________________ |

**14. Who do you think should deliver a self-management support program? (check one response only)**

|  | Health care professional(s) such as a nurse, rehabilitation specialist |
| --- | --- |
|  | Non-health care professionals |
|  | Individual(s) who has/have a traumatic spinal cord injury |
|  | Individual(s) who has/have a neurological condition (e.g., multiple sclerosis) |
|  | Caregiver of an individual with a traumatic spinal cord injury |
|  | Combination of any of above choices (Specify): ______________________ |
|  | Other, please specify... ______________________ |

**15. Who do you think should be responsible for organizing the self-management program (e.g., informing patients about the existence of the program, training staff to deliver the program, advertising the program)? (check one response only)**

|  | Family physician |
| --- | --- |
|  | Physiatrist |
|  | Neurologist |
|  | Case manager |
|  | Staff in the acute care team |
|  | Staff in the rehabilitation team |
|  | An organization such as the Canadian Paraplegic Association |
|  | Staff in the home care team |
|  | Other, please specify... ______________________ |

**16. How important is it for a self-management program to be developed for individuals with traumatic spinal cord injury?**

|  | Very Unimportant |
| --- | --- |
|  | Unimportant |
|  | Neither Important or Unimportant |
|  | Important |
|  | Very Important |

**PART III – Consumer Characteristics**

**1. Moorong Self-Efficacy Scale (MSES) (Middleton et al., 2003)**

|  | 1=Very Uncertain | 2 | 3 | 4 | 5 | 6 | 7=Very Certain |
| --- | --- | --- | --- | --- | --- | --- | --- |
| 1. I can maintain my personal hygiene with or without help. |  |  |  |  |  |  |  |
| 2. I can avoid having bowel accidents. |  |  |  |  |  |  |  |
| 3. I can participate as an active member of the household. |  |  |  |  |  |  |  |
| 4. I can maintain relationships in my family. |  |  |  |  |  |  |  |
| 5. I can get out of my house whenever I need to. |  |  |  |  |  |  |  |
| 6. I can have a satisfying sexual relationship. |  |  |  |  |  |  |  |
| 7. I can enjoy spending time with my friends. |  |  |  |  |  |  |  |
| 8. I can find hobbies and leisure pursuits that interest me. |  |  |  |  |  |  |  |
| 9. I can maintain contact with people who are important to me. |  |  |  |  |  |  |  |
| 10. I can deal with unexpected problems that come up in life. |  |  |  |  |  |  |  |
| 11. I can imagine being able to work at some time in the future. |  |  |  |  |  |  |  |
| 12. I can accomplish most things I set out to do. |  |  |  |  |  |  |  |
| 13. When trying to learn something new, I will persist until I am successful. |  |  |  |  |  |  |  |
| 14. When I see someone I would like to meet, I am able to make the first contact. |  |  |  |  |  |  |  |
| 15. I can maintain good health and well-being. |  |  |  |  |  |  |  |
| 16. I can imagine having a fulfilling lifestyle in the future. |  |  |  |  |  |  |  |

**2. Which of the following elements of self-management are you already incorporating in your daily life to reduce health problems and/or increase well-being? (check all that apply)**

|  | Engaging in exercise |
| --- | --- |
|  | Practicing pain and fatigue management |
|  | Practicing relaxation techniques (e.g., guided imagery and breathing exercises) |
|  | Dealing with depression |
|  | Learning about nutrition |
|  | Communicating with family |
|  | Communicating with health care professionals |
|  | Practicing problem solving |
|  | Practicing goal setting/action planning |
|  | Other, please specify... ______________________ |

**3. Mastery or Locus of Control (Pearlin and Schooler Mastery Scale, 1978)**

Now a series of statements that people might use to describe themselves. Please tell me if you strongly agree, agree, neither agree nor disagree, disagree, or strongly disagree.

|  | Strongly Agree | Agree | Neutral | Disagree | Strongly Disagree |
| --- | --- | --- | --- | --- | --- |
| 1. You have little control over the things that happen to you. |  |  |  |  |  |
| 2. There is really no way you can solve some of the problems you have. |  |  |  |  |  |
| 3. There is little you can do to change many of the important things in your life. |  |  |  |  |  |
| 4. You often feel helpless in dealing with problems of life. |  |  |  |  |  |
| 5. Sometimes you feel that you are being pushed around in life. |  |  |  |  |  |
| 6. What happens to you in the future mostly depends on you. |  |  |  |  |  |
| 7. You can do just about anything you really set your mind to. |  |  |  |  |  |

**4. Patient Activation Measure (Hibbard et al., 2005)**

Below are some statements that people sometimes make when they talk about their health.  Please indicate how much you agree or disagree with each statement as it applies to you personally by circling your answer.  Your answers should be what is true for you and not just what you think others want you to say.If the statement does not apply to you, click N/A.

|  | Disagree Strongly | Disagree | Agree | Agree Strongly | N/A |
| --- | --- | --- | --- | --- | --- |
| 1. When all is said and done, I am the person who is responsible for taking care of my health |  |  |  |  |  |
| 2. Taking an active role in my own health care is the most important thing that affects my health |  |  |  |  |  |
| 3. I am confident I can help prevent or reduce problems associated with my health |  |  |  |  |  |
| 4. I know what each of my prescribed medications do |  |  |  |  |  |
| 5. I am confident that I can tell whether I need to go to the doctor or whether I can take care of a health problem myself |  |  |  |  |  |
| 6. I am confident that I can tell a doctor concerns I have even when he or she does not ask |  |  |  |  |  |
| 7. I am confident that I can follow through on medical treatments I may need to do at home |  |  |  |  |  |
| 8. I understand my health problems and what causes them |  |  |  |  |  |
| 9. I know what treatments are available for my health problems |  |  |  |  |  |
| 10. I have been able to maintain (keep up with) lifestyle changes, like eating right or exercising |  |  |  |  |  |
| 11. I know how to prevent problems with my health |  |  |  |  |  |
| 12. I am confident I can figure out solutions when new problems arise with my health |  |  |  |  |  |
| 13. I am confident that I can maintain lifestyle changes, like eating right and exercising, even during times of stress |  |  |  |  |  |

**Insignia Health. "Patient Activation Measure; Copyright © 2003-2010, University of Oregon. All Rights reserved"**

**5. Hospital Anxiety and Depression Scale (HADS) (Zigmond & Snaith, 1983)**

Read each item below and click or mark the reply which comes closest to how you have been feeling in the past week.Don’t take too long over your replies, your immediate reaction to each item will probably be more accurate than a long, thought-out response.

|  | Most of the time | A lot of the time | From time to time, occasionally | Not at all |
| --- | --- | --- | --- | --- |
| I feel tense or ‘wound up’ |  |  |  |  |
|  | Definitely as much | Not quite so much | Only a little | Hardly at all |
| I still enjoy the things I used to enjoy |  |  |  |  |
|  | Very definitely and quite badly | Yes, but not too badly | A little, but it doesn’t worry me | Not at all |
| I get a sort of frightened feeling as if something awful is about to happen |  |  |  |  |
|  | As much as I always could | Not quite so much now | Definitely not so much now | Not at all |
| I can laugh and see the funny side of things |  |  |  |  |
|  | A great deal of the time | A lot of the time | Not too often | Very little |
| Worrying thoughts go through my mind |  |  |  |  |
|  | Never | Not often | Sometimes | Most of the time |
| I feel cheerful |  |  |  |  |
|  | Definitely | Usually | Not often | Not at all |
| I can sit at ease and feel relaxed |  |  |  |  |
|  | Nearly all the time | Very often | Sometimes | Not at all |
| I feel as if I am slowed down |  |  |  |  |
|  | Not at all | Occasionally | Quite often | Very often |
| I get a sort of frightened feeling like ‘butterflies’ in the stomach |  |  |  |  |
|  | Definitely | I don’t take as much care as I should | I may not take quite as much care | I take just as much care as ever |
| I have lost interest in my appearance |  |  |  |  |
|  | Very much indeed | Quite a lot | Not very much | Not at all |
| I feel restless as if I have to be on the move |  |  |  |  |
|  | As much as I ever did | Rather less than I used to | Definitely less than I used to | Hardly at all |
| I look forward with enjoyment to things |  |  |  |  |
|  | Very often indeed | Quite often | Not very often | Not at all |
| I get sudden feelings of panic |  |  |  |  |
|  | Often | Sometimes | Not often | Very seldom |
| I can enjoy a good book or radio or television programme |  |  |  |  |

**HADS copyright © R.P. Snaith and A.S. Zigmond, 1983, 1992, 1994. Record form items originally published in Acta Psychiatrica Scandinavica, 67, 361–70, copyright © Munksgaard International Publishers Ltd, Copenhagen, 1983. This edition first published in 1994 by nferNelson Publishing Company Ltd. GL Assessment, 9th Floor East, 389 Chiswick High Road, London W4 4AL. GL Assessment is part of the GL Education Group. This form may not be reproduced by any means without first obtaining permission from the publisher. Email: permissions@gl-assessment.co.uk**

**6. Cognitive Symptom Management (Stanford Chronic Disease Self-Management Study, 1996)**

When you are feeling down in the dumps, feeling pain or having other unpleasant symptoms, how often do you (please identify one for each question):

|  | Never | Almost Never | Sometimes | Fairly Often | Very Often | Always |
| --- | --- | --- | --- | --- | --- | --- |
| 1. Try to feel distant from the discomfort and pretend that it is not part of your body. |  |  |  |  |  |  |
| 2. Don’t think of it as discomfort but as some other sensation, like a warm, numb feeling. |  |  |  |  |  |  |
| 3. Play mental games or sing songs to keep your mind off the discomfort. |  |  |  |  |  |  |
| 4. Practice progressive muscle relaxation. |  |  |  |  |  |  |
| 5. Talk to yourself in positive ways. |  |  |  |  |  |  |

**7. Communication with Physician Scale (Stanford Chronic Disease Self-Management Study, 1996)**

When you visit your doctor, how often do you do the following (please identify one for each question):

|  | Never | Almost Never | Sometimes | Fairly Often | Very Often | Always |
| --- | --- | --- | --- | --- | --- | --- |
| 1. Prepare a list of questions for your doctor |  |  |  |  |  |  |
| 2. Ask questions about the things you want to know and thing you don’t understand about your treatment. |  |  |  |  |  |  |
| 3. Discuss any personal problems that may be related to your illness. |  |  |  |  |  |  |

**THANK YOU VERY MUCH!!! Your participation in spinal cord injury research is important.  People who work in the field of spinal cord injury use the information to provide better care now and in the future. If you have any questions or concerns regarding the survey, you can contact Sarah Munce at sarah.munce@utoronto.ca or at toll-free at1-855-946-7902.**

**Please send us your address so that we can mail you your $10.00 gift certificate.**
